# Succoring the challenging acute mesenteric ischemia: Feasibility of lactate dehydrogenase for evaluation of intestinal necrosis extension and mortality

**DOI:** 10.1016/j.amsu.2022.104922

**Published:** 2022-11-17

**Authors:** Danny Michell Conde Monroy, Felipe Girón Arango, Lina Rodríguez Moreno, Carlos Eduardo Rey Chaves, Andrea Donoso-Samper, Ricardo Nassar, Andrés Isaza-Restrepo

**Affiliations:** a School of Medicine, Universidad del Rosario, Bogotá, Colombia; b Department of Surgery, Hospital Universitario Méderi, Bogotá, Colombia; c Department of Surgery, Hospital Universitario Fundación Santa Fe de Bogotá, Bogotá, Colombia; d School of Medicine, Universidad de los Andes, Bogotá, 110111, Colombia; e School of Medicine, Pontifical Xavierian University, Bogotá, Colombia

**Keywords:** Mesenteric ischemia, Lactate, Surgery

## Abstract

**Background:**

Acute mesenteric ischemia is a lethal challenging pathology for surgeons in the emergency department due to its ambiguous clinical presentation and lack of early diagnostic markers. Serum lactate is considered a relevant biomarker in terms of bowel necrosis length and mortality prediction. Nevertheless, its association has been poorly studied. Hence, we evaluated the relation between serum lactate admission levels, bowel necrosis extension, and mortality in patients with acute mesenteric ischemia.

**Methods:**

A Retrospective cross-sectional study with a prospective database was conducted, including patients over 18 years old with mesenteric ischemia that required surgical management between January 2012 and December 2018. We describe the association between serum lactate admission levels with bowel necrosis length and mortality in patients with acute mesenteric ischemia.

**Results:**

74 patients presented with acute mesenteric ischemia, 44 males and 30 females. Mean age was 73.5 ± 10.7 years old. Significant association between serum lactate admission levels and mortality was found (ROC cut-value of 3.8 mmol/l, 81.0% sensibility and 76% specificity, LR+3.41 (95%CI 1.57, 7.40), LR- 0.25 (95%CI 0.13–0.45))(P.001). Nonetheless no statistically significant association was found between serum lactate admission levels and bowel necrosis length (ρ = 0.195,95%CI -0.046, −0.436, P > .99). As post hoc analysis, a classification and regression tree on mortality was fitted.

**Conclusions:**

Early diagnosis, prognosis and management of mesenteric ischemia is vital given its high morbidity and mortality. Serum lactate admission levels can be considered as a useful prognostic tool in terms of mortality in patients with acute mesenteric ischemia.

## Highlights

• Lactate dehydrogenase has been studied as a biomarker for acute mesenteric ischemia.
• Association between lactate dehydrogenase, bowel necrosis length, and mortality.
• A classification and regression tree calculated mortality profiles and predictors.
• No association between lactate dehydrogenase and bowel necrosis length was found.
• Lactate dehydrogenase of 3.8 mmol/l was the most important predictor of mortality.

## 1 Background

Acute mesenteric ischemia (AMI) is a challenging pathology for general surgeons in the emergency department due to its ambiguous clinical presentation and lack of precocious tools for diagnosis [1]. It is considered a vascular emergency secondary to a sudden interruption of small intestine blood supply, that can lead to an ominous outcome even if treated [1,2]. AMI can be classified as occlusive (OAMI) or non-occlusive (NOMI) in terms of its etiology, being OAMI associated with 60–80% of all cases [1,[3], [4], [5], [6], [7], [8]].

Despite being a rare entity with an incidence of 0.09–0.2% of all admissions to the emergency department and 1–2% of gastrointestinal illnesses, its suspicion and diagnosis must be prompt due to its high mortality rate (32–92%) [1,[4], [5], [6], [7], [8], [9], [10], [11], [12], [13]]. Intestinal ischemia stems from transmural necrosis of the bowel wall caused by severe hypoperfusion, which can progress to sepsis, peritonitis or extensive gangrene [1,[4], [5], [6]]. Initial management includes gastrointestinal decompression, fluid resuscitation, hemodynamic monitoring and support, correction of electrolyte abnormalities, pain control, anticoagulation under most circumstances, and initiation of broad-spectrum antibiotics [1,[4], [5], [6],^10^,^13^]. Despite this, surgery should not be delayed in patients suspected of having intestinal infarction or perforation based upon clinical, radiographic, or laboratory parameters, regardless of etiology [1,[4], [5], [6],^10^,^13^,^14^]. In cases who present with peritonitis or obvious bowel perforation, the confirmatory diagnosis will necessarily be made in the operating room [10,13,14].

On the other hand, delayed surgical management might lead to larger extension of bowel necrosis, requiring larger resections with the subsequent undesired consequences such as short bowel syndrome, long-term parenteral nutrition and detriment to life quality [1,4,14]. Even though multiple markers such as serum lactate [2,12,[14], [15], [16], [17], [18], [19], [20], [21]], l-lactate [[20], [21], [22], [23], [24], [25]], d-dimer [26,27] and intestinal fatty acid-binding protein (I-FABP) [[28], [29], [30], [31]] have been employed to ease AMI diagnosis, none has shown accurate and consistent results [1,4,5,7]. Lack of a reliable marker for prediction of bowel necrosis extension and mortality leads to surgical procedures where inoperable massive bowel infarction is evidenced [14,16,24]. Serum lactate is a frequently used hypoperfusion biomarker, it is inexpensive and available in most centers, but results in most studies show heterogeneous sensitivity and specificity in AMI [2]. Given the growing demand of tools that help elucidate diagnosis, bowel compromise and mortality, we aim to describe the association between serum lactate in the emergency room, bowel necrosis extension and mortality.

## 2 Methods

With the Institutional Review Board's approval, following Health Insurance Portability and Accountability Act (HIPAA) guidelines, a retrospective review of a prospectively collected database was conducted. All patients over 18 years of age that required laparotomy with a postoperative confirmed diagnosis of AMI were included between january 2012 and december 2018. Patients with no description of the serum lactate admission levels (SLAL) or extension of intestinal necrosis were excluded. The present study has been reported in line with STROCCS guidelines [32] Ethical compliance with the Helsinki Declaration, current legislation on research Res. 008430-1993 and Res. 2378-2008 (Colombia) and the International Committee of Medical Journal Editors (ICMJE) were ensured under our Ethics and Research Institutional Committee (IRB) approval.

Preoperative data included patient demographics, comorbidities, symptoms, findings in the physical examination, serum lactate admission levels, blood analysis results, CT results. Intraoperative and postoperative data included surgical findings, pathology report of intestinal necrosis and 30 days mortality. Data was reviewed by external investigators from UR-SIG, a research group alliance forged by Universidad del Rosario and Hospital Universitario Mayor de Mederi to evaluate data quality.

Descriptive statistics were reported in terms of variable nature. Qualitative analysis was performed in terms of frequencies and percentages, while quantitative analysis was done in terms of mean and standard deviations of normally distributed data and medians and interquartile ranges (IQRs) for non-normally distributed data. Bivariate analysis was performed. Qualitative variables were analyzed using chi-square statistics (Fisher's exact test when appropriate). Quantitative variables were analyzed, based on normality, with Spearman's or Pearson's associations correlation coefficient accordingly. Bivariate analysis between qualitative and quantitative variables was performed using Mann-Whitney test or the *t*-test for independent samples [32,33]. For associations between categorical variables, odds ratios with 95% confidence intervals were provided. Diagnostic performance of SLAL for mortality was evaluated using the receiver operating characteristic curve (ROC) [[32], [33], [34]].

Classification and regression tree (CART) [32] implemented in the R package *part* was fitted to assess the predictive power of relevant sociodemographic, clinical, and laboratory variables for mortality. A multivariable logistic regression model was fitted with the highest importance value variables selected by the CART model without any mathematical transformation. Finally, the ROC curve of the decision tree was calculated. For both ROC curves, the area under de ROC curve (AUC) with its 95% confidence interval is reported [35]. Positive likelihood ratio (LR+) and negative likelihood ratio (LR-) with their 95% confidence intervals were calculated [36]. Specificity and sensibility were reported with their 95% exact binomial confidence limits. Statistical analysis was performed using R Software 3.6.3.39.

## 3 Results

### 3.1 Descriptive statistics

From January 2012 to December 2018 a total of 74 patients underwent urgent laparotomy with a postoperative diagnosis of AMI. Mean age was 73.5 ± 10.7 years old. 44 Males and 30 females. Mean body mass index was 25 ± 2.9 kg/m2 (Table 1). 15 patients (20%) presented NOMI. All the patients presented abdominal pain, 17 (23%) had peritoneal signs and 23 (31%) gastrointestinal bleeding on physical examination. Median time from symptom's onset to arrival to the emergency room (ER) was 24 (IQRs 61) hours. Median SLAL was 5.6 (IQRs 5) mmol/l. Median time between AMI's diagnosis and surgical management was 5 (IQRs 5) hours. Documented bowel necrosis involved the small intestine and colon with a median length of 161.5 (IQRs 207) cm. Surgical resection was performed in 37 (50%) patients. Overall, mortality within thirty days was 72%, of which 35 occurred within the first 24 postoperative hours.

**Table 1:** Descriptive statistics.

Sociodemographic Characteristics (n = 74)	No (%)
Men	44 (60)
Age, mean (SD), y	73.5 (10.7)
Body Mass Index, mean (SD), kg/m2 (n = 39)	25 (2.9)
**Comorbidities (n = 74)**	**No (%)**
Smoking tobacco	16 (22)
Alcohol consumption	7 (10)
Hypertension	50 (68)
Diabetes mellitus type II	17 (23)
Peripheral vascular disease	14 (19)
Chronic kidney disease	12 (16)
Atrial fibrillation	12 (16)
COPD	14 (19)
Coronary heart disease	10 (14)
**Admission Characteristics (n = 74)**	**No (%)**
Time of symptoms onset on arrival to the emergency room, median (IQRs), h	24 (61)
Abdominal pain	74 (100)
Peritoneal signs	17 (23)
Gastrointestinal bleeding	23 (31)
Multiorgan failure	54 (75)
SLAL, median (IQRs), mmol/L	5.6 (5)
Total WBC, median (IQRs), 106/mm3	14555 (18270)
Ph, mean (SD)	7.31 (0.1)
Base excess, mean (SD)	−9.4 (6.6)
CPR, median (IQRs), mg/L	71.6 (151)
Marshall Score	
2	19 (29)
3	12 (18)
4	9 (12)
1	7 (9)
6	5 (7)
7	5 (7)
8	4 (6)
9	3 (4)
5	2 (3)
**CT-Scan Findings (n = 28)**	**No (%)**
Bowel Dilatation	20 (69)
Mesenteric arterial or venous obstruction	17 (53)
Ascites	12 (43)
Decreased Bowel Enhancement	7 (25)
Pneumatosis intestinalis	3 (11)
Pneumoperitoneum	1 (3)
**Surgical Findings and Outcomes (n = 74)**	**No (%)**
Time from diagnosis to surgery, median (IQRs), h	5 (5)
Bowel Necrosis Length, median (IQRs), cm	161.5 (207)
Bowel resection	37 (50)
NOMI	15 (20)
Vessel Occlusion	
Venous	11 (19)
Arterial	48 (81)
Death within 30 postsurgical days	53 (72)
Postsurgical death day, median (IQRs), d	1 (1)
Death on the first postsurgical day	35 (66)
**Pathology Findings n = 37**	**No (%)**
Bowel Necrosis Length in pathology, median (IQRs), cm	105 (101)
Acute inflammation	32 (84)
Cellular necrosis in pathology	32 (84)
Transmural hemorrhage in pathology	30 (78)

COPD: Chronic obstructive pulmonary disease, SLAL: Serum lactate admission levels, WBC: White blood cells, CPR: c-reactive protein, CT: Computed tomography, NOMI: non-obstructive mesenteric ischemia.

### 3.2 Analytic statistics of serum lactate admission levels (SLAL), bowel necrosis length, and mortality

**Fig. 1:**
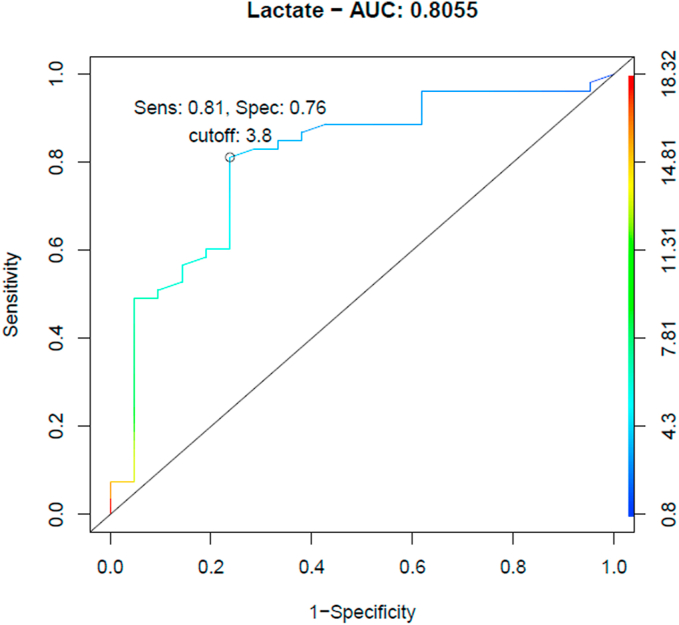
ROC Curve for mortality prognosis of serum lactate admission levels. Colored scale corresponds to SLAL threshold values. Area under the curve 0.8055, 95% CI 0.6901–0.9209, LR+ 3.41 (1.57–7.40), LR- 0.25 (0.13–0.45).

Non-significant statistical association between SLAL and necrosis length was established (ρ = 0.195, 95%CI -0.046 to −0.436, P > .99) (Table 2). Median SLAL in fatal cases was 6.3 (IQRs 4.5) mmol/l and 2.9 (IQRs 1.3) mmol/l (P.001) in non fatal (Table 3). SLAL cut-value for mortality of 3.8 mmol/l was determined by ROC- analysis with a sensibility of 81.0% (95% CI: 68–91%) and specificity of 76% (95% CI: 53–92%), LR+ 3.41 (95% CI: 1.57–7.40), LR- 0.25 (95% CI: 0.13–0.45) (Fig. 1).

**Table 2:** Serum lactate admission levels- cross-tabulation.

	Median (IQRs)	Effect measure (95%CI)	p-value*
Primary outcomes			
Bowel necrosis length, correlation	–	0.195 (-0.04–0.43)	>.99
Death within 30 postsurgical days			
Yes	6.3 (4.5)	–	.001
No	2.9 (1.3)	–	
**Secondary outcomes**			
Men	5.9 (5.3)	–	>.99
Women	4.3 (4.8)	–	
Age, correlation	–	−0.032 (-0.277–0.214)	>.99
Body Mass Index, correlation	–	0.138 (-0.169–−0.443)	>.99
**Comorbidities**			
Smoking tobacco			
Yes	6 (5.6)	–	.>.99
No	4.9 (4.9)	–	
Alcohol consumption			
Yes	5.1 (26.3)	–	>.99
No	5.8 (5)	–	
Hypertension			
Yes	6 (5.3)	–	>.99
No	5 (3.5)	–	
Diabetes mellitus type II			
Yes	4 (4.2)	–	>.99
No	6 (5.1)	–	
Peripheral vascular disease			
Yes	7.4 (2.7)	–	>.99
No	4.8 (5)	–	
Chronic kidney disease			
Yes	6.6 (8)	–	>.99
No	5.3 (5)	–	
Atrial fibrillation			
Yes	6.6 (5.1)	–	>.99
No	5.2 (5.1)	–	
COPD			
Yes	6.5 (6)	–	>.99
No	5 (5)	–	
Coronary heart disease			
Yes	5.4 (5.8)	–	>.99
No	5.6 (5)	–	
**Admission Characteristics**			
Time of symptoms onset on arrival to the emergency room, correlation	–	−0.052 (-0.16–0.4)	>.99
Peritoneal signs			
Yes	6 (3)	–	.>.99
No	5.1 (5.3)	–	
Gastrointestinal bleeding			
Yes	6 (9.1)	–	>.99
No	4.9 (5)	–	
Multiorgan failure			
Yes	5.2 (4.9)	–	>.99
No	6.9 (5.6)	–	
Total WBC, correlation	–	0.15 (-0.08–0.3)	>.99
Ph, correlation	–	−0.5 (-0.7–−0.3)	<.001
Base excess, correlation	–	−0.63 (-0.784–−4.78)	<.001
CPR, correlation	–	0.135 (-0.4–0.708)	>.99
Marshall Score			
1	5.4 (5.3)	–	>.99
2	6.8 (3.8)	–	
3	2.9 (5.2)	–	
4	5 (3.3)	–	
5	4.7 (3.8)	–	
6	4.9 (2.2)	–	
7	7 (1.8)	–	
8	8.3 (3.8)	–	
9	5 (4.1)	–	
**CT-Scan Findings**			
Bowel Dilatation, mean (SD			
Yes	5.4 (3.6)	(Reference)	>.99
No	5.9 (4.4)	0.5 (-2.7–3.8)	
Mesenteric arterial or venous obstruction			
Yes	3.7 (5.4)	–	>.99
No	5.8 (3.8)	–	
Ascites, mean (SD)			
Yes	5.4 (3.6)	(Reference)	>.99
No	6 (4)	0.5 (-2.3–3.4)	
Decreased Bowel Enhancement, mean (SD)			
Yes	5.7 (3.6)	(Reference)	>.99
No	5.7 (3.9)	0.014 (-3.1–3.2)	
Pneumatosis intestinalis, mean (SD)			
Yes	8.8 (5.4)	–	>.99
No	4.3 (4.6)	–	
Pneumoperitoneum			
Yes	14.9 (0)	–	>.99
No	4.7 (4.7)	–	
**Surgical Findings and Outcomes**			
Time from diagnosis to surgery, correlation	–	−0.274 (-0.5–−0.04)	.74
Bowel resection			
Yes	4.8 (4.5)	–	>.99
No	6 (5.5)	–	
NOMI			
Yes	4 (2.1)	–	>.99
No	6.1 (5.4)	–	
Vessel Occlusion			
Arterial	7 (4.2)		.001
Venous	2.8 (0.6)		
Postsurgical death day, correlation	–	−0.407 (-0.6–−0.1)	.095
Death on the first postsurgical day			
Yes	4.15 (3)	–	.14
No	7.8 (4.2)	–	
**Pathology Findings**			
Bowel Necrosis Length in pathology, correlation	–	−0.101 (-0.4–0.22)	>.99
Acute Inflammation			
Yes	4.75 (4)	–	>.99
No	8.6 (7)	–	
Cellular necrosis in pathology			
Yes	5.9 (5.8)	–	>.99
No	4 (0.9)	–	
Transmural hemorrhage in pathology			
Yes	4.5 (4.9)	–	>.99

COPD: Chronic obstructive pulmonary disease, SLAL: Serum lactate admission levels, WBC: White blood cells, CPR: c-reactive protein, CT: Computed tomography, NOMI: non-obstructive mesenteric ischemia. Effect measure corresponds to mean difference and its 95% confidence interval, estimated by *t*-test if normality assumption held, otherwise median and IQRs are reported. For continuous variables spearman correlation coefficient is reported with its 95% confidence interval. * p-value corrected with the Bonferroni method.

**Table 3:** Mortality at 30 post-surgical days- Cross-Tabulation.

	Non-Survivorsn = 53 (72) n (%)	Survivorsn = 21 (28) n (%)	Effect measure (95%CI)	p-value*
Men	29 (55)	15 (71)	1 (Reference)	>.99
Women	24 (45)	6 (29)	0.49 (0.1–1.4)	
Age, mean (SD)	74 (10)	71 (12)	−3.28 (-9.1–2.5)	>.99
Body Mass Index, mean (SD)	25 (2.9)	23 (2.4)	−3.32 (-28–21)	>.99
**Comorbidities**				
Smoking tobacco				
Yes	12 (23)	4 (19)	1 (Reference)	>.99
No	41 (77)	17 (81)	0.9 (0.3–3.9)	
Alcohol consumption				
Yes	3 (6)	4 (19)	1 (Reference)	>.99
No	50 (94)	17 (81)	0.2 (0.06–1.2)	
Hypertension				
Yes	37 (70)	13 (62)	1 (Reference)	>.99
No	16 (30)	8 (38)	1.4 (0.4–4.7)	
Diabetes mellitus type II				
Yes	12 (23)	5 (24)	1 (Reference)	>.99
No	41 (77)	16 (76)	0.7 (0.2–2.8)	
Peripheral vascular disease				
Yes	12 (23)	2 (10)	1 (Reference)	>.99
No	41 (77)	19 (90)	1.8 (0.5–10.1)	
Chronic kidney disease				
Yes	11 (21)	1 (5)	1 (Reference)	>.99
No	42 (79)	20 (95)	2.5 (0.6–21.9)	
Atrial fibrillation				
Yes	9 (17)	3 (14)	1 (Reference)	>.99
No	44 (83)	18 (85)	0.9 (0.2–4.3)	
COPD				
Yes	10 (19)	4 (19)	1 (Reference)	>.99
No	43 (81)	17 (81)	0.7 (0.2–3.2)	
Coronary heart disease				
Yes	5 (9)	5 (24)	1 (Reference)	. >.99
No	48 (91)	16 (76)	0.2 (0.09–1.2)	
**Admission Characteristics**				
Time of symptoms onset on arrival to the emergency room, median (IQRs)	24 (60)	24 (61)	–	>.99
Peritoneal signs				
Yes	11 (21)	6 (29)	1 (Reference)	>.99
No	42 (79)	15 (71)	0.5 (0.21–1.9)	
Gastrointestinal bleeding				
Yes	18 (34)	5 (24)	1 (Reference)	>.99
No	35 (66)	16 (76)	1.6 (0.5–5.7)	
Multiorgan failure				
Yes	42 (81)	12 (60)	1 (Reference)	>.99
No	10 (19)	8 (40)	2.7 (0.8–8.7)	
SLAL, median (IQRs)	6.3 (4.5)	2.9 (1.3)	–	.001
Total WBC, median (IQRs)	14740 (6830)	12790 (8800)	–	>.99
Ph, mean (SD)	7.2 (0.14)	7.4 (0.08)	0.131 (0.06–0.2)	<.001
Base excess, median (IQRs)	−11.1 (-15.2, −6.6)	−6.4 (-5.5)	–	>.99
CPR, mean (SD)	156.2 (139)	38 (48)	−117.5 (-210–−24)	>.99
Marshall Score				
1	3 (6.5%)	4 (20)	3 (0.3–238)	>.99
2	11 (23.9%)	8 (40)	2 (0.2–114)	
3	8 (17.3%)	4 (20)	1.3 (0.1–88)	
4	5 (10.8%)	4 (20)	2 (0.23–142)	
5	2 (4.3%)	0	0 (0.02–97)	
6	5 (10.8%)	0	0 (0.01–40)	
7	5 (10.8%)	0	0 (0.01–40)	
8	4 (8.6%)	0	0 (0.012–49)	
9	3 (6.5%)	0	1 (Reference)	
**CT-Scan Findings**	**n = n(%)**	**n = n(%)**		
Bowel Dilatation				
Yes	14 (74)	6 (60)	1 (Reference)	>.99
No	5 (26)	4 (40)	1.3 (0.3–8.6)	
Mesenteric arterial or venous obstruction				
Yes	12 (55)	5 (50)	1 (Reference)	>.99
No	10 (45)	5 (50)	0.9 (0.2–5)	
Ascites				
Yes	7 (37)	5 (55)	1 (Reference)	>.99
No	12 (63)	4 (44)	0.3 (0.1–2.2)	
Decreased Bowel Enhancement				
Yes	4 (21)	3 (33)	1 (Reference)	>.99
No	15 (79)	6 (67)	0.3 (0.1–2.8)	
Pneumatosis intestinalis				
Yes	3 (16)	0 (0)	1 (Reference)	>.99
No	16 (84)	9 (100)	1.5 (0.1–86)	
Pneumoperitoneum				
Yes	1 (5)	0 (0)	1 (Reference)	>.99
No	18 (95)	10 (100)	0.5 (0.0–6.45)	
**Surgical Findings and Outcomes**				
Time from diagnosis to surgery, median (IQRs)	4 (4)	5.5 (8)	–	>.99
Bowel necrosis length, median (IQRs)	258 (273)	100 (100)	–	.16
Bowel resection				
Yes	22 (42)	15 (71)	1 (Reference)	>.99
No	31 (58)	6 (29)	0.2 (0.09–0.85)	
NOMI				
Yes	11 (21)	4 (19)	1 (Reference)	>.99
No	42 (79)	17 (21)	0.87 (0.31–3.5)	
Vessel Occlusion				
Arterial	39 (93)	9 (53)	1 (Reference)	.054
Venous	3 (7)	8 (47)	7.8 (2.4,42)	
Postsurgical death day, median (IQRs)	1 (1)	2 (0)	−1.7 (-14.3–−10.8)	>.99
Death on the first postsurgical day				
Yes	35 (67)	0 (0)	1 (Reference)	>.99
No	17 (33)	1 (100)	0 (0.006–4)	
**Pathology Findings**				
Bowel Necrosis Length in pathology, median (IQRs)	110 (100)	75 (107)	–	>.99
Acute Inflammation				
Yes	18 (78)	14 (93)	1 (Reference)	>.99
No	5 (22)	1 (6)	0.2 (0.05–2)	
Cellular necrosis in pathology				
Yes	18 (78)	14 (93)	1 (Reference)	>.99
No	5 (22)	1 (6)	0.2 (0.05–2)	
Transmural hemorrhage in pathology				
Yes	18 (78)	12 (80)	1 (Reference)	>.99
No	5 (22)	3 (20)	0.69 (0.2–4.3)	

COPD: Chronic obstructive pulmonary disease, SLAL: Serum lactate admission levels, WBC: White blood cells, CPR: c-reactive protein, CT: Computed tomography, NOMI: non-obstructive mesenteric ischemia. Effect measure corresponds to odds ratios for categorical variables and mean difference for continuous variables if normality assumption held, otherwise medians and IQRs are reported. *p-value corrected by the Bonferroni method.

### 3.3 Classification and regression tree (CART) and logistic regression

**Fig. 2:**
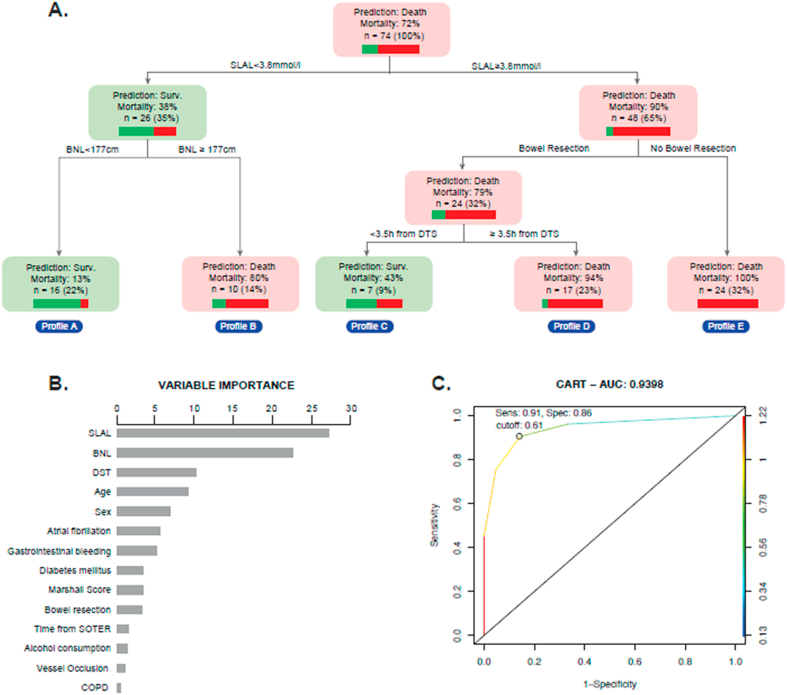
Acute mesenteric ischemia: mortality of the classification and regression tree (CART). SLAL: serum lactate admission levels, BNL: Bowel necrosis length, DTS: Diagnosis to surgery SOTER: symptoms onset to emergency room arrival. COPD: Chronic Obstructive Pulmonary Disease **A**. Classification and regression tree. **B.** Variable importance bar chart. C. ROC Curve on the prediction of mortality of CART. Colored scale corresponds to the predicted probability of dying. Cut-value of dying probability were 0.61 with sensibility 91% and specificity 86%, LR+ 6.34 (2.22–18.14), LR- 0.11 (0.05–0.26). Area under the curve 0.93 (95%CI 0.8869–0.9927).

CART was fitted to determine the importance and cutoff value of variables on mortality (Fig. 2A), resulting variables were included in the logistic regression (Table 4). The variables with the highest importance value were SLAL with a cut-value of 3.8 mmol/l, bowel necrosis length with a cut-value of 177 cm, time of performance of surgical procedure within 3.5 h after diagnosis, and bowel resection (Fig. 2B). A ROC curve showed a cut-value of 0.61 on the probability of the mortality with 91% sensibility (95% CI: 79–97%) and 86% specificity (95% CI: 64–97%), LR+ 6.34 (95% CI: 2.22–18.14), LR- 0.11 (95% CI: 0.05–0.26) (Fig. 2C).

**Table 4:** Logistic regression analysis on risk factors for mortality.

Variable	AOR (95%CI)	P-value
(Intercept)	0.006 (0.00–0.97)	.059
Serum lactate	1.53 (1.20–2.15)	.003
Age	1.04 (0.98–1.12)	.13
Sex, Women	2.60 (0.66–11.54)	.18
Bowel Necrosis Length	1.00 (0.99–1.01)	.22
Bowel Resection	0.44 (0.10–1.95)	.28
Time from diagnosis to surgery	0.98 (0.93–1.04)	.5

AOR: adjusted odds ratio, 95% CI: 95% confidence interval P-value: p-value of Wald test for significance of regression term.

## 4 Discussion

In this retrospective cross-sectional study of patients with AMI who underwent laparotomy, non statistically significant correlation between SLAL and bowel necrosis length was documented despite reported in literature by different studies [15,18]. Nonetheless, a statistically significant correlation between SLAL and mortality was elucidated. SLAL cut-value of 3.8 mmol/l for mortality prediction was identified with a sensitivity of 81% and specificity of 76%, LR+ 3.41 (1.57, 7.40), LR- 0.25 (0.13–0.45), which relates to results found by Leone et al. (cut-value of 3.9 mmol/l, sensitivity of 60% and a specificity of 83%) [16] and Caluwaerts et al. (cut value of 3.65 mmol/l) [17]. CART analysis showed SLAL had the highest importance value (27%) in predicting mortality and was the only significant variable in the logistic regression in relation with former studies that have shown serum lactate as an important independent risk factor for mortality [15,17,37].

The CART model provided five mortality profiles (Fig. 2) that may be relevant in terms of prognosis. Predictions were done using a SLAL cut-off point of 3.8 mmol/l, from there, mortality was determined by bowel necrosis length, intestinal resection and time within surgical procedure was performed. Cases with a necrosis length shorter than 177 cm had a 13% mortality, while those with longer necrosis had a mortality of 80%, in concordance with reports by Akyıldız et al. in a retrospective study of 104 patients with AMI, where an association (OR 5.6, p = .002) between necrosis length (>100 cm) and mortality was found [15,38,39].

Prompt diagnosis and surgical management associated with bowel resection constitutes an important factor associated with fatal outcomes in patients with mesenteric ischemia [16,40,41]. In our study, these variables are shown to be determinant factors in the CART model, resembling results reported by Kassahun et al. and Park et al. Kassahun et al. described that intestinal viability is maintained in 100% of patients with symptoms that lasted less than 12 h compared to only 20% viability in those with symptoms that lasted longer than 24 h [41], while Park et al. showed that bowel resection at first or second-look procedure decreased the mortality rate with a relative risk ratio of 0.5 (95%CI, 0.2–0.9) [11].

According to our model, an based on a high suspicion of AMI, in patients with an SLAL below 3.8 mmol/l, survival could be determined by the bowel necrosis length, while for cases with SLAL over 3.8 mmol/l, survival could be determined by a bowel resection performed within 3.5 h after diagnosis. Thus, SLAL might be a potential mortality biomarker for AMI and an objective tool for a patient's prognosis. Stemming from this, our CART model might be a reliable tool to characterize a patient's mortality risk, with an ROC-Curve cut-value of 0.61 on the probability of the mortality's threshold (91% sensibility, 86% specificity, LR+ 6.34 (2.22,18.14) and LR- 0.11 (0.05,0.26)). Nevertheless, the surgical team must consider different described variables to align preoperative and postoperative management and most importantly discuss prognosis with the patient and his family.

It is important to take into account that despite SLAL, bowel necrosis length, bowel resection, and the time from diagnosis to surgery appear relevant, only SLAL is a mortality marker in all cases. This difference might be explained by the fact that latter variables are not crucial for the entire population but only for patients with specific profiles shown in the CART. For instance, bowel necrosis length is relevant for patients with SLAL below 3.8 mmol/l but not for those with higher SLAL values. Hence, the CART model is a valuable statistical tool that classifies the population into subgroups and identifies crucial variables for each one, which has never been conducted before regarding AMI research.

On the other hand, our mortality rate was 72%, higher in contrast to other studies [2,11,15,16,18,42,43]. Differences could be explained by the median time from symptom onset to arrival to the emergency room in our population (24 (IQRs 61) hours). Upon arrival at the emergency room, all the subjects presented abdominal pain while gastrointestinal bleeding and peritonitis signs had a low incidence, which concurs with the classical clinical description of AMI and previously reported data [11,43]. Common comorbidity factors associated with this entity, like diabetes mellitus, arterial hypertension, atrial fibrillation, and peripheral vascular disease, had similar prevalence as reported in other studies [2,11,15,18,43].

In spite of the similar results of vague clinical findings and clinical diagnosis relying on a high suspicion index found in our cases and in the literature, imaging is considered a helpful tool that can be used after careful consideration of time available [1,4,6]. A computed tomography (CT) scan has a 93% sensitivity and 100% specificity for AMI [1]. In this study, only 38% of cases had a CT-Scan done. Patients with a high clinical suspicion or non immediate availability of CT-Scan were taken directly to surgery. Bowel dilatation was the main finding on the CT-Scan (69%) similar to those reported by Nuzzo et al., followed by mesenteric obstruction (53%) (arterial or venous), ascites (43%), and decrease bowel enhancement 25%) [2]. Once diagnosis is made, treatment must be established, being laparotomy the gold-standard for this pathology [1,6], nonetheless, several case series have proposed endovascular revascularization procedure as an alternative to AMI in patients without evidence of bowel ischemia or infarction [[44], [45], [46]].

On the whole, mesenteric ischemia diagnosis is reached through a combination of clinical, laboratory, and imaging findings, which must be optimized in order to perform a surgical or angiology intervention as early as possible [1,45]. Our study suggests that SLAL may be a relevant marker for mortality in AMI and that the decision tree proposed might guide identification, prognosis, and management.

Among the limitations of this study are its retrospective nature, non-systematic measurement of serum lactate levels according to symptoms onset but only on arrival to the emergency department and biased serum lactate levels due to sepsis, shock, impaired liver or kidney function, exposure to toxins, diabetes, or malignancies [37,47].

## 5 Conclusion

SLAL and bowel necrosis length did not evidence a statistically significant correlation. However, SLAL had the highest importance value in the prediction of mortality using CART with 5 different profiles. Implementation of this new tool, can provide a feasible instrument for prognostic expectations. Nonetheless, given our work limitations, more studies are needed to replicate and validate these results.

## Statements

The authors have no relevant financial or non-financial interests to disclose.

The authors have no competing interests to declare that are relevant to the content of this article.

All authors certify that they have no affiliations with or involvement in any organization or entity with any financial interest or non-financial interest in the subject matter or materials discussed in this manuscript.

The authors have no financial or proprietary interests in any material discussed in this article.

## Ethical approval

Ethical approval was reached.

## Sources of funding

None declared.

## Author contribution

D.C Research idea.

D.C, F.G, L.R, A.D Data analysis, manuscript writing.

D.C,F.G, L.R, A.D, A.I, R.N: Manuscript writing, edition and critical review.

D.C Final revision of the manuscript.

## Registration of research

1. Name of the registry:
2. Unique Identifying number or registration ID:
3. Hyperlink to your specific registration (must be publicly accessible and will be checked):

## Guarantor

Danny Conde.

## Provenance and peer review

Not commissioned, externally peer-reviewed.

## Declaration of competing interest

None declared.
